# Supplementary material for: Identification of photoperiod-induced specific miRNAs in the adrenal glands of Sunite sheep (Ovis aries)
Source: Front Vet Sci. 2022 Jul 22;9:888207. doi: 10.3389/fvets.2022.888207 (PMC9354845; doi:10.3389/fvets.2022.888207)
Supplement: Supplementary file 1 [file Data_Sheet_1.ZIP › Supplementary file/miRDeep2 file.zip!/miRDeep2 file/Novel_Structure/SPLP42A1/Novel_502.pdf]

Provisional ID	:	NC_019484.2_32630
Score total	:	1.6
Score for star read(s)	:	-1.3
Score for read counts	:	0
Score for mfe	:	1.3
Score for randfold	:	1.6
Score for cons. seed	:	
Total read count	:	63
Mature read count	:	63
Loop read count	:	0
Star read count	:	0

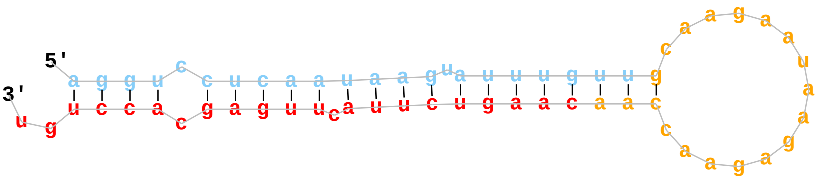

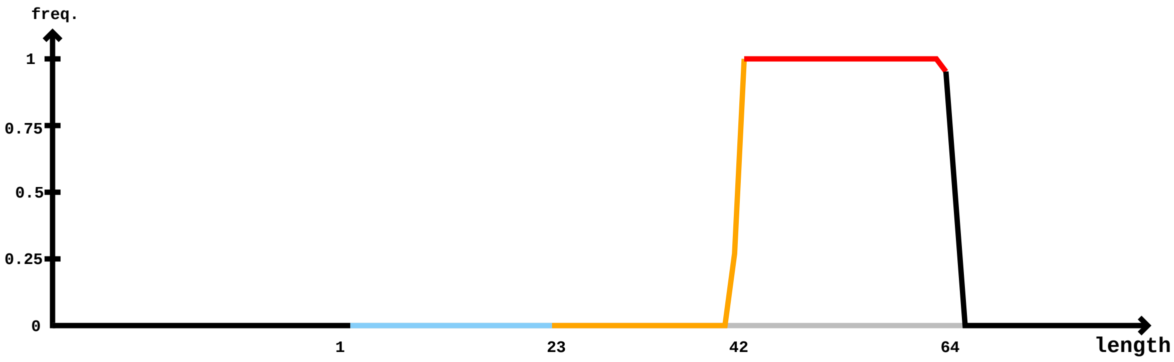


```
                                   Star                                        Mature
5'- auuguguuucuucaugccugacauauagcagguccucaauaaguauuuguugcaagaauaagagaaccaacaagucuuacuugagcaccuguuauguggaggauaaaaagcu   -3'    exp
    .....((((.((.((.(((..((((((((((((.(((((((((.((((((((...............)))))))))))).))))).)))))))))))).))))))).)))).    reads      mm         sample
    .....................................................................acaagucuuacuugagcaccug.....................    2          0          seq
    .....................................................................acaagucuuacuugagcaccugu....................    11         0          seq
    .....................................................................acaagucuuacuugagcaccuguu...................    4          0          seq
    ......................................................................caagucuuacuugagcaccug.....................    1          0          seq
    ......................................................................caagucuuacuugagcaccugu....................    36         0          seq
    ......................................................................caagucuuacuugagcaccuguu...................    9          0          seq
```
